# Balancing economic and social results in football clubs: evidence of fans’ perceptions in the Colombian context

**DOI:** 10.3389/fspor.2025.1541829

**Published:** 2025-04-07

**Authors:** Juan Alejandro Hernández-Hernández, Abraham Londoño-Pineda, Robert Ng-Henao, Juan Lucas Macías-Franco, Jose Alejandro Cano

**Affiliations:** Faculty of Economic and Administrative Sciences, University of Medellin, Medellin, Colombia

**Keywords:** actives fans, non-active fans, fan pressures, economic and social outcomes, cocreation of value, Colombian football

## Abstract

Fans are not only loyal, but also powerful, and could therefore exert pressure on football club managers. This study examines the influence of fan-generated pressure on the economic and social sustainability outcomes of football clubs in Colombia, highlighting the role of active and non-active fan typologies. Using a conceptual framework rooted in stakeholder theory, we developed and tested a structural equation model (SEM) based on Colombian football fans. Results show that active fans, defined as direct or indirect followers, prioritize high-performing players and coaches and significantly influence economic outcomes through merchandising and broadcasting rights. Conversely, non-active fans, including passive and potential supporters, emphasize player stability and club goodwill. These findings highlight that fan pressure, shaped by sporting identity and performance expectations, serves as a critical lever for value co-creation and sustainability.

## 1 Introduction

In different social contexts, the prevailing perspective rooted in the “shareholder supremacy” developed by Friedman (1), maintains that the primary responsibility of managers is to maximize shareholder profits, operating under the premise that increased corporate earnings directly translate to greater wealth accumulation. This explains why companies' business models have traditionally been oriented towards the creation of value, mainly for their shareholders (2). In contrast, the stakeholder perspective developed by Freeman et al. (3), challenges the traditional profit-maximization paradigm, arguing that companies should transcend narrow shareholder interests and embrace a more comprehensive approach to value creation that encompasses the broader ecosystem of organizational stakeholders.

In this regard, a framework for applying stakeholder theories to the sport industry has been developed by McCullough and Cunningham (4), who argue that managers are motivated to include stakeholders in their business strategy primarily because of the pressures they generate. In the specific case of football, evidence has been found that club managers choose to include their stakeholders in their business objectives based on the pressures they face (5–7). In the specific case of football, institutional stakeholders have been considered by McCullough et al. (8), and societal and market stakeholders such as football players, coaches, fans and communities have also been mentioned by Todaro et al. (7).

While all stakeholders play a key role in the football industry, it is only with fans that the so-called fan's affective connection exists (9, 10). This means that fans identify with the clubs they support to such an extent that this feeling leads them to develop a high degree of loyalty, which in turn creates a long-term relationship with the club and, in economic terms, can represent demand for the goods and services offered by the club (11, 12). Therefore, ignoring fan-generated pressure could have serious consequences for football club finances (13, 14), justifying the choice of fans as the football stakeholders on which this study will focus.

Most of the studies that include stakeholder pressure have two characteristics: they have mainly focused on European leagues and they have been oriented towards the environmental component of sustainability, as can be seen in the works of Fernández-Villarino (15), Daddi et al. (6), Cayolla et al. (16), Todaro (7), among others. However, there is no evidence on the role of pressure exerted by different types of supporters on the economic and social outcomes of football teams in South America, but particularly in Colombia (17). It should be noted that in Colombia, football culture is particularly rooted in different social groups (18) Colombia even exports quality players to other international leagues. In fact, between 2017 and 2022, Colombia became the third largest exporter of players in South America, after Argentina and Brazil, and the fifth largest exporter of players in the world (19), which shows the importance of this type of research in this country.

## 2 Framework

The basis for more recent work on the typology of stakeholders associated with football has been developed by McCullough and Cunningham (4) and McCullough et al. (8), who have proposed a framework based on institutional theory. In this theory, institutions are represented by formal and informal norms, i.e., both laws and systems of values and beliefs embedded in society. In this sense, the concept of institutional stakeholders has gained currency (5). Precisely, in the work of Todaro et al. (7), these institutional stakeholders were classified as governmental stakeholders, football institutions, and market and societal stakeholders. Governmental stakeholders refer to governments at different levels (municipal, departmental, national), football institutions represent the actors that manage the football sector with their regulations and tournaments at different levels (FIFA, UEFA, Conmebol, etc.), while market and societal stakeholders include suppliers, sponsors, fans and local communities.

As this is an evolving field, classifications of football stakeholders are not abundant (5, 15). However, this study considers as a basis the disaggregation of institutional stakeholders (government stakeholders, football institutions, market stakeholders and society), specifically, fans. Football is a particular sport in which the commitment of the fans to their clubs must be taken into account, since the passion they feel makes them develop a sense of loyalty that turns them into long-term consumers of the services offered by the teams they follow (11, 12). This indicates a connection between fans and their clubs, which has been conceptualized as the affective connection of fans (15, 16, 20). The implications of this affective connection legitimize fans as a crucial stakeholder in football. It generates an impact on football clubs' sources of income, such as stadium attendance, sales of clothing and souvenirs, and the payment of affiliation fees to sports channels, which represent the broadcasting rights of football teams, among others (21).

Furthermore, the existence of the affective connection of fans as well as the relationship with fans influences the long-term financial sustainability of football clubs. Therefore, it is necessary for football organizations to understand the behavior of their fans and their needs in order to implement actions that satisfy the sporting performance and empower fans in the club's activities, programs and objectives, resulting in the potential to generate more profit and co-create value (22). Consequently, this theoretical framework is intended to provide the basis for the elaboration of the conceptual model that supports the measurement of the SEM, based on the characterization of fan typologies, the characterization of identity and sport performance pressures, and the characterization of the expected economic and social outcomes that these pressures generate.

### 2.1 Fan typologies

Pioneering academic works and research that laid the foundation for fan typologies are those of Mitchell and Wood (23) and Senaux (21), who provided three criteria for classifying fans to explain their different levels of identity: legitimacy, power, and sense of urgency. Legitimacy is understood as a desirable social good that is broader and more shared than a simple self-perception and therefore helps to identify stakeholders that deserve management attention, i.e., it indicates which stakeholders really count (23). In the specific case of football, fans of football teams are legitimate in the sense that they have the power to impose their will in relation to what they consider urgent (19). In this sense, legitimacy is related to aspects such as the trust that fans have in the club, which is derived from loyalty and long-term commitment (7, 11, 20).

Power, on the other hand, is based on demand that affects the club's finances in the short or long term. These actions include attending football matches, paying for broadcasting rights, purchasing services, and club sport's sponsorships (24, 25). In the case of the sense of urgency, it stands out the most among highly passionate fans, particularly when sporting performance declines, recruitment strategies appear inadequate, or club management seems to lack a coherent sports vision. These dedicated fans are most likely to critique club leadership and express their dissatisfaction, demonstrating an intense commitment that transcends casual loyalty (26–28).

The classification of football fans is essential for understanding how different supporter groups influence club management and sustainability outcomes. This study adopts a streamlined typology focused on active fans (direct fans, indirect fans), and non-active fans (passive fans, and potential fans), aligning with previous research while ensuring relevance to the proposed conceptual model (19).

#### 2.1.1 Active fans

Active fans engage regularly with the football club, either through physical attendance at matches, as direct fans do, or by following the club remotely through digital platforms as indirect fans do (29). Direct fans demonstrate the highest level of engagement, attending matches through single or season ticket purchases (30). Their loyalty to the club is strong, contributing to financial sustainability through stadium attendance and merchandise sales, and exerting strong performance-related pressure, demanding managerial decisions that enhance sporting success (7, 20, 27, 31). In the case of indirect fans, while they are not physically present at matches, they follow the club through broadcasting channels, streaming services, or social media (32, 33). Their sense of urgency varies, but they still influence club strategies, generating revenue for the club through digital subscriptions and media rights, particularly regarding media-driven engagement (30, 34).

#### 2.1.2 Non-active fans

Non-active fans may not engage directly with the club, but they can still impact club decision-making, particularly in areas of reputation and corporate social responsibility (17). Passive fans demonstrate a low level of club loyalty and follow the team infrequently, without making significant financial contributions (20, 27). While they may consume football content, their level of urgency and influence on club governance is limited. In contrast, potential fans, while not traditional supporters, can develop an attachment to a club through social responsibility initiatives (16). These individuals may be drawn to sustainability programs, gender equality in sports, or environmental campaigns (15, 35). Some scholars refer to them as green consumers, highlighting their preference for clubs that align with broader social values (36).

**Figure 1:**
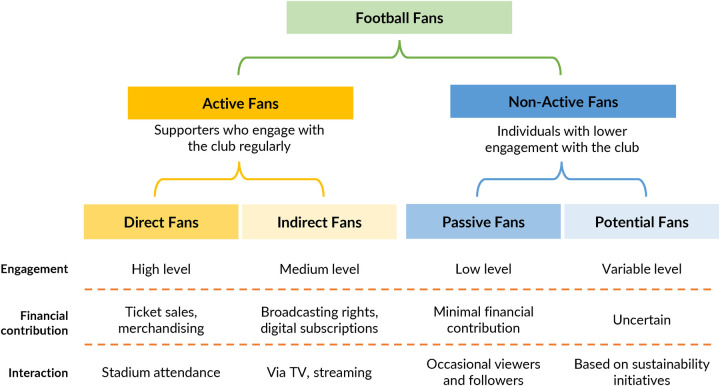
Football fan typologies.

Figure 1 provides a summary of these fan typologies, which form the foundation for linking fan pressures to economic and social outcomes within football clubs.

### 2.2 Pressures of identity and sporting performance

The literature identifies a number of pressures that derive from the concept of isomorphism, which is understood as standardized or homogenized behavior of firms that derives from social norms and behaviors (4). In this regard, DiMaggio and Powell (37), propose the existence of three types of pressures that make up the conceptualization of isomorphism: coercive, mimetic and normative. Coercive pressures are those that are mandatory, or those that are not mandatory but become common practice in the sector to which the organization belongs or by society, leading firms to adapt to these types of requirements. These are the most common in the first wave of environmental sustainability practices (38). This type of pressure comes mainly from governmental stakeholders or football institutions, as these are the ones that have the greatest impact on the normativity (5–7). Mimetic pressures, on the other hand, have more to do with football organizations, as they tend to imitate the behavior of their peers (8), so it is common for them to have developed during the second wave of sustainability practices (17). Finally, there are normative pressures that are more related to the diffusion of educational institutions (39, 40), which refer to customs, traditions and social behaviors. In the specific case of football, the latter are more related to market and social actors, such as local communities and fans (7).

While the three typologies mentioned above underline the importance of behaviors and social norms, they do not show the tensions arising from the traditional pyramidal structure that prevails in the football sector, which in many cases pits the interests of the moral owners of football teams (fans) against the legal owners (shareholders, entrepreneurs) (41). In this sense, de Witte and Zglinski (42) have made some conceptual contributions to structuring the pressures stemming from the cultural elements of the sport model, composed of identity commitment and sport performance. Identity commitment in football clubs is intrinsically linked to local cultural traditions (43) and strategic communication processes that reduce information asymmetries with stakeholders, particularly fans. Recognizing that fan loyalty is central to club management (26), emerging research suggests that football clubs' business models should prioritize the positive emotional engagement of their supporter base (31, 44). Moreover, according to Meier et al. (45), managers and owners of clubs should be fans of the clubs they belong to, and to improve club management, fans should also be full or partial owners of the clubs' fans.

Another component of pressure is the focus on sporting success. It is important for fans that their clubs strive for sporting success by winning local and international matches and tournaments (46). Fans feel part of the club due to the affective connection they have with it, so the sporting defeats of the football clubs are taken as their own (20). One of the ways to achieve sporting performance is for managers to hire high performing players (27), as well as a high performing coaching staff, as coaches are the face of the club and are easily the target of fans' joy or frustration with the club's performance (47). Therefore, the pressures associated with regional identity are related to four variables that make up the sporting identity and performance pressures in this study. They are represented by the sense of belonging of the football club's managers (9, 45), the recruitment of high performing players (48, 49), the stability of the player squad (50), and the recruitment of a high performing coaching (47, 51).

Football fan protests represent an important form of coercive pressure, with supporters actively challenging club management and governing bodies over economic, social and even political issues (5). This activism is often fueled by concerns over ticket pricing, fair competition, and governance integrity, reinforcing the role of fans as key stakeholders in the football ecosystem (52). Protests can range from social media campaigns to large-scale demonstrations during globally televised matches, illustrating how fans operate as collective cultural critics and co-authors of the club as a cultural institution (53). These protests can highlight the negative impact of club and government decisions, particularly in relation to commercialization, financial mismanagement and the social responsibility of football institutions (4). Historically, fan groups have played a visible role in social movements, as demonstrated by the Gezi Park protests in Turkey and the 2014 Bosnian uprisings, where football fans collectively mobilized in political and non-political agitation (54, 55). Beyond direct political engagement, protests in football have also emerged in response to the increasing commercialisation of the sport, reflecting fan discontent with rising ticket prices, the financial priorities of club owners, and a perceived disconnect from traditional football values (55, 56). Some supporters have gone as far as establishing independent, community-owned clubs as a form of resistance, seeking to reclaim the sport from corporate interests (57).

In this paper, the characterization of fan-generated pressures on football club managers suggests that the main fan-generated pressures are related to identity commitment and sporting performance. Putra (43), argues that football club identity fundamentally connects with local traditions and regional heritage, positioning football as a dynamic social space where collective local identities are actively constructed and reinforced. In this perspective, managers' sense of belonging to the club is seen as a reflection of their commitment to the club's identity (17), even club managers should be fans of the club, as they understand the sufferings and joys of the fans (45).

### 2.3 Impact of pressures on the economic and social performance of football clubs

Transitioning from a shareholder-centric value creation model to a stakeholder-inclusive approach represents a fundamental reimagining of football clubs' business strategies, emphasizing collaborative and holistic value generation (17). In this context, several authors have mentioned different forms of co-creation. Zagnoli and Radicchi (22) point out that fan communities are not spectators, but active and necessary stakeholders in joint value creation. Castro-Martinez and Jackson (58) highlight the importance of linking football objectives with commercial objectives, referring to the co-creation of social and economic value. Similarly, Bull and Whittam (41) propose a useful framework based on the creation of sustainable value, concluding that there is excessive economic exploitation of the owners of English football clubs to the detriment of the social and cultural capital represented by the moral owners of the club (the fans), straining the relationship with them. Fernández-Villarino (15) and Lozano and Barreiro-Gen (35), argue that the football industry must recognize fan engagement as a critical strategic dimension, rooted in the profound emotional attachment supporters have with their clubs. This shared identity presents a compelling opportunity for collaborative value creation, ultimately manifesting in the clubs' sustainability outcomes.

Daddi et al. (6) proposed a model in which a number of pressures generate effects on environmental performance outcomes, such as environmental governance practices and environmental operational practices. They included the environmental dimension but did not consider the other dimensions of sustainability as the focus of their work was on the environment. Todaro et al. (7) present a model that includes pressures from different stakeholders such as government interest groups, football institutions and market and societal stakeholders such as local communities, fans and sponsors. In their research, the pressures from these stakeholders affect performance outcomes, which are reflected in four expected benefits: internal management, environmental performance, reputation and goodwill, and corporate sponsorship. In the work of Cayolla et al. (16), fan pressure influences a set of performance outcomes that the authors call social, environmental and economic benefits.

The focus of the present work is that the pressure generated by different types of fans has an impact on the different expected outcomes of football clubs (17). Thus, in terms of economic results, fans can affect the income from ticket sales and season tickets (28, 30), the sale of goods and services (merchandising) of the football club (13, 14), as well as the income that clubs receive from broadcasting rights (27). In terms of social outcomes, fans pressure manifests itself in aspects such as goodwill and reputation (6, 7, 16), or the club's sporting performance (46), although the intensity of these pressures depends on the type of the fans that generates them.

Both active and non-active fans have the potential to influence the financial performance of football clubs, as they can generate income from individual and season tickets (59), by purchasing merchandising (9, 10), or by paying subscriptions to sports channels (27). However, active fans, especially direct fans, are more likely to attend the stadium and purchase goods and services offered by the club (30), while indirect fans may be more likely to follow the club through broadcasting channels (32). In the case of non-active fans, the most common means by which they would follow the club would be through broadcasting channels (17). As it concerns the evaluation of fans and its overall impact on economic outcomes, Hypothesis 1 is stated as follows:

*H1: The ratings given by fans indicate that the identity and sporting performance-based pressures have a significant impact on the economic outcomes of football clubs*.

In terms of social outcomes, the identity and sporting performance pressures generated by all fan typologies should have a positive impact on reputation and goodwill (reputation). At least, this has been demonstrated by studies on the pressures generated by different stakeholders in terms of goodwill and reputation (7, 16). However, it could be that active fans are more interested in the club's sporting performance due to a higher emotional attachment (15, 45), but since it concerns the evaluation of fans to overall social outcomes, Hypothesis 1 is formulated as follows:

*H2: The ratings given by fans indicate that identity and sporting performance-based pressures have a significant impact on the social outcomes of football clubs*.

**Figure 2:**
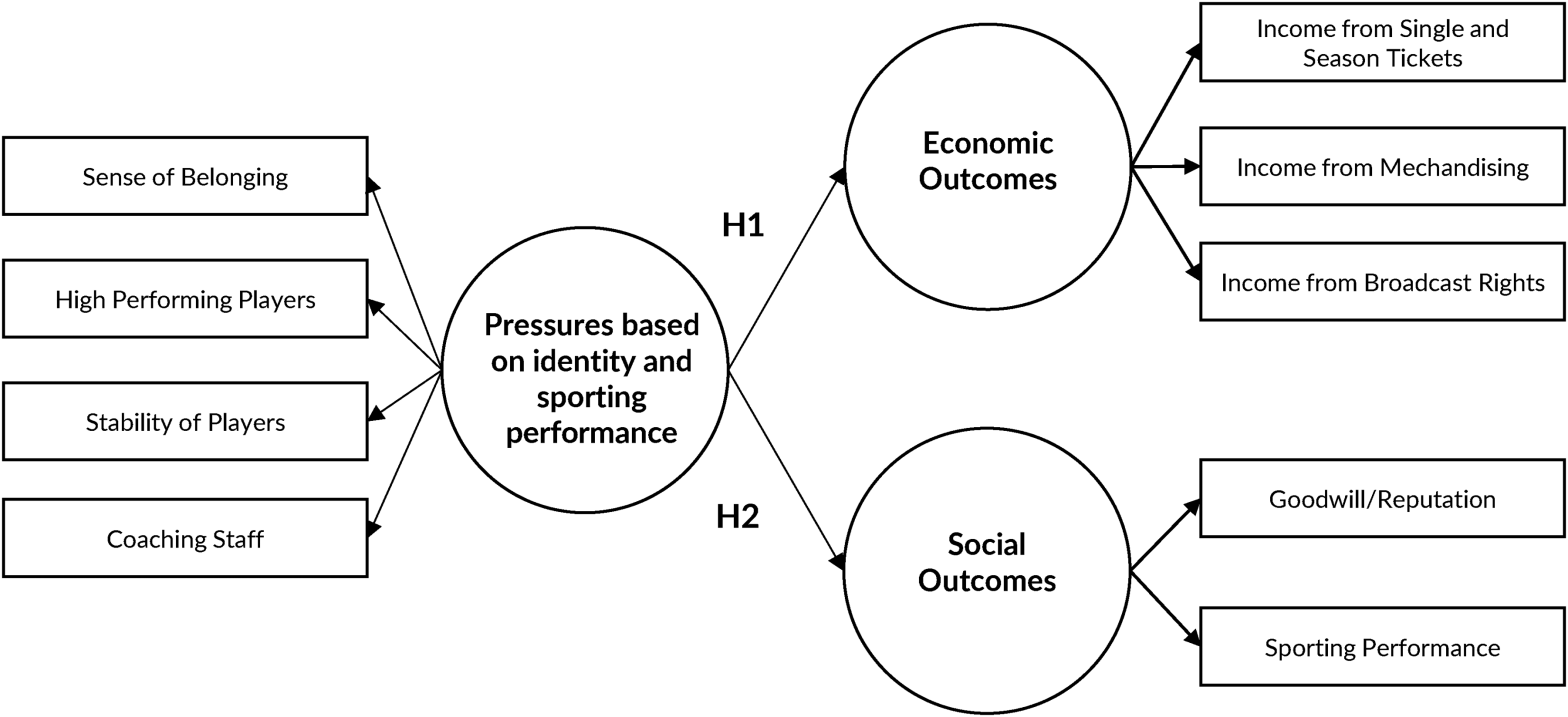
Conceptual model.

Therefore, the conceptual model proposed from the theoretical framework is shown in Figure 2, which relates fan pressures based on identity and sporting performance to the economic and social outcomes of football clubs.

## 3 Methodology

The ownership structure of football clubs in Colombia reflects a diverse mix of private investment, family ownership, and business group control, which significantly influences club management and financial decision-making. Unlike the membership-based club model in Brazil and Argentina, where fans often play a role in club governance, Colombian clubs are primarily owned by private investors, business conglomerates, and wealthy families. In Colombia's first division league, Liga BetPlay Dimayor, several clubs are controlled by large business groups. For example, Atlético Nacional is owned by Organización Ardila Lulle, a large Colombian business conglomerate, while Millonarios F.C. is controlled primarily by the investment firm Amber Capital, which also has interests in European clubs such as Lens (France) and Real Zaragoza (Spain). Similarly, Junior de Barranquilla is owned by the influential Char family, which has extensive investments in retail, media and sports.

Other clubs, such as América de Cali and Independiente Santa Fe, are majority owned by individual investors such as Tulio Gómez and Diego Perdomo, respectively. Some clubs, such as Deportivo Independiente Medellín, operate under a joint-stock company model in which shareholders participate in the management of the club. In addition, recent trends show the entry of foreign investors, such as La Equidad, which was acquired by a U.S.-based investment group that includes actors Ryan Reynolds, Rob McElhenney and Eva Longoria. Similarly, Fortaleza F.C. has shareholders such as Colombian singer Carlos Vives and cyclist Rigoberto Urán. This privatized and investor-driven structure impacts club decision-making, financial sustainability, and long-term planning, and influences player recruitment, sponsorship deals, and overall club strategies, shaping how teams respond to economic and fan-driven pressures. This context provides essential background for analyzing the relationship between fan engagement, financial pressures, and club management in Colombia.

The aim of this study is to examine the influence of the pressures of identity and sporting performance that fans generate on the expected results of the football clubs they support and follow. To this purpose, a questionnaire (survey) was administered to 604 people (Colombian fans), and respondents were informed that their personal data would not be disclosed as the purpose of the survey was purely academic. Before applying the questionnaire to the respondents, the questions were validated through a qualitative study based on the criteria of experts belonging to the two main categories: active and non-active fans. The answers given by these types of fans served as the basis for formulating the hypotheses to be tested in the SEM. To obtain responses from respondents, an electronic form was made available on Google Forms and distributed through a network of collaborators involved in research on fan pressure in football. The form is aimed at all types of fans and does not differentiate by gender or age to be representative of different types of fans and different perceptions of football club performance.

After receiving the completed forms, responses for those who declared themselves not to be fans of any football team and had no interest in being a fan were discarded, resulting in a final number of 501 responses for the study. The respondents were divided into two general categories, active and non-active fans, based on a question aimed at categorizing the respondents according to the identified fan typology. The four options of football fan identity represent the following categories: Direct fans, indirect fans, passive fans, and potential fans. Active fans are made up of direct, and indirect fans (29), while non-active fans consists of passive fans (27, 32) and potential fans (16). Within the sample size, 17.0% (85) of respondents were categorized as direct fans, 34.3% (172) as indirect fans, 41.3% (207) as passive fans, and 7.4% (37) as potential fans.

The second section of the survey included questions on the four variables related to the pressure based on identity and sporting performance, namely the sense of belonging, the high-performing players, the stability of the players, and the coaching staff. For each variable, a scenario was presented in which the different economic and social outcomes were asked about. To illustrate, in the economic dimension, questions were asked about stadium attendance, purchases of merchandise, and subscriptions to sports channels. Similarly, in the social dimension, questions focused on the impact on goodwill and sporting performance. Each response option was presented on a Likert scale: 1—strongly disagree, 2—disagree, 3—neither agree nor disagree, 4—agree, and 5—strongly agree. Table 1 shows the questions asked of the different types of fans, related to the identity and sporting performance pressures and their influence on the expected economic and social outcomes of football clubs.

**Table 1:** Questions for each type of pressure and expected sustainability outcomes.

Pressures based on identity and sporting performance	Questions	Economic			Social	
		Income from single and season tickets	Income from merchandising	Income from sports broadcasting rights	Reputation or Goodwill	Sport Performance
Sense of belonging of managers	1. If the management of the football club of which you are a fan shows a sense of belonging, then:	You would attend the stadium by buying single and season tickets	You would buy the football club's sporting goods and accessories (clothing, caps, jackets, T-shirts, shorts, key rings, etc.)	You would pay for subscriptions to television channels that broadcast football matches	You think that this situation would damage the club's reputation or goodwill	You think that this situation would improve the club's sporting performance
High-performance players	2. If the management of the football club of which you are a fan hires high performance players, then:					
Stability of player squad	3. If the management of the football club of which you are a fan guarantees the stability of players, then:					
Coaching staff	4. If the management of the football club of which you are a fan guarantees a High Technical Staff, then:					

For the proposed SEM, identity and sporting performance pressures represent the first-order construct variable, and it is made up of the following indicators: managers' sense of belonging, high-performing players, stability of the players squad, and high-performing coaching staff. The economic and social outcomes of football clubs represent the second-order construct variables. The economic outcomes construct is made up of the following indicators: income from individual and season tickets, income from merchandising, and income from broadcasting rights. Likewise, the social outcomes construct is made up of the following indicators: goodwill and sporting performance. All the observed variables that make up the construct variables are defined as reflective. Table 2 shows the description of the variables that make up the proposed SEM.

**Table 2:** Variables for the SEM.

Construct variables	Observed variables	Abbreviation	Description	Calculation method
Pressures based on identity and sporting performance	Sense of belonging	VAL-SoB	Represents the fans’ valuation of sense of belonging.	Average of the questions related to sense of belonging
	High performing players	VAL-HPP	Represents the fans’ valuation of high-performing players.	Average of the questions related to high-performance players.
	Stability of players	VAL-SoP	Represents the valuation of the fans towards the stability of players.	Average of the questions related to stability of players.
	Coaching staff	VAL-TS	Represents the valuation of the fans regarding the coaching staff.	Average of the questions related to the coaching staff.
Economic Outcomes	Income from single and season tickets	TIC	Represent the fans’ valuation of income generation caused by solidarity-based pressures.	Average of the questions related to income from single and seasons tickets
	Income from mechandising	MER		Average of the questions related to merchandising income
	Income from broadcasting rights	BR		Average of the questions related to broadcasting rights income
Social Outcomes	Goodwill	GW	Represent the fans’ valuation of the social impact caused by solidarity-based pressures.	Average of the questions related to goodwill
	Sporting performance	SP		Average of the questions related to sporting performance

SEM can be approached through variance-based SEM (CB-SEM) and variance-based SEM (PLS-SEM) techniques. CB-SEM is primarily used for factor-based models, where the goal is to confirm or test existing theories by estimating a covariance matrix for the sample data (60, 61). In contrast, PLS-SEM is more appropriate for composite-based models that focus on predicting key constructs and exploring relationships between variables that cannot be measured directly (62). PLS-SEM was chosen for this study because it supports both formative and reflective measurement models, whereas CB-SEM is limited to reflective models only (61). This flexibility is particularly relevant given the study's aim to examine the pressures exerted by different fan typologies on the economic and social outcomes of football clubs, which involve constructs that are best represented as composites rather than latent factors. In addition, PLS-SEM is better suited to exploratory modeling and theory extension, making it ideal for this study, which aims to identify key relationships rather than confirm an existing theoretical framework (60).

In this regard, we employed partial least squares structural equation modeling (PLS-SEM), as a sophisticated multivariate analysis technique increasingly prominent in management research (63). Unlike traditional statistical methods, this approach enables a comprehensive investigation of complex relationships among observed and latent variables from a predictive perspective. Notably, PLS-SEM allows for the modeling of structural paths with formative constructs without imposing restrictive distributional assumptions on the data, thereby providing greater analytical flexibility (64). In addition, PLS-SEM operates similarly to multiple regression analysis, allowing for the estimation of complex relationships with relatively small sample sizes while maintaining high predictive accuracy. Given these methodological considerations, PLS-SEM provides a robust approach to analyzing the impact of fan pressure on football club sustainability, ensuring both statistical reliability and practical applicability. The implementation of the PLS-SEM analysis is supported by the SmartPLS 4 software, which stands out for its robust and user-friendly capabilities, multivariate analysis features, easy model visualization and result representation through graphical outputs, and its widespread adoption in advanced research.

The study's distinctive approach involves examining the research model across two distinct fan groups, active and non-active fans, to comprehensively analyze the pressures within different fan segments. This approach ensures greater sample representativity while maintaining analytical clarity. A more segmented model, differentiating direct, indirect, passive, and potential fans individually, would result in redundant findings and an unnecessarily complex discussion. By grouping fans into these two broader categories, the analysis remains focused on meaningful distinctions in fan influence while avoiding excessive repetition. To achieve this analytical depth, the hypotheses presented in the conceptual model are tested for active fans and non-active fans.

## 4 Results

The final dataset for this study consists of 501 responses from Colombian football fans, with a demographic distribution that matches the general characteristics of football fans in the region. Most respondents are young adults aged 18–24 (58.7%), followed by individuals aged 25–34 (14.6%) and 35–44 (12.0%). This age distribution reflects the dominant fan base of football clubs, which tends to be younger and more actively involved in the sport. In terms of gender, the sample includes 60.3% male and 39.7% female respondents, demonstrating a significant level of female participation, in line with the growing interest of women in football fandom.

Geographically, responses were received from 40 different municipalities across Colombia, with a strong representation from Medellín (62.5%), followed by Bello (9.8%), Envigado (5.8%), Itagüí (4.0%) and Sabaneta (2.6%). This regional concentration corresponds to the strong presence of two of Colombia's most popular football clubs, Atlético Nacional (63.7%) and Independiente Medellín (18.8%), both based in Medellín. The sample's club affiliations are representative of national trends, as Atlético Nacional has the largest fan base in Colombia and Independiente Medellín is the second most followed club in Medellín. The dataset also includes fans of other major Colombian clubs such as América de Cali, Junior F.C. and Millonarios F.C., ensuring a diverse representation.

Moreover, the results of this study also include both the measurement and the structural model, as recommended by Marrucci et al. (65), that are presented as follows.

### 4.1 Measurement model

The path coefficients are useful for testing the hypothesized model because they indicate how much the predictor variables contribute to explaining the variance of the endogenous variable (66). According to Bhatt (67), these coefficients are significant if they are greater than 0.2. However, according to Hoe (68), to be more rigorous, they should be greater than 0.3. Similarly, to check the reliability of the constructs, the Cronbach Alpha analysis is required, and according to Nunnally (69), it should be greater than 0.7. The structured evaluation of the model must also be checked, which is done using R-squares and should be greater than 0.1 according to Miles (70). Similarly, to check the internal consistency of the model, an analysis of the average variance extracted (AVE) should be carried out, which is only applicable to reflective variables, and according to Chin (71), this value should be greater than 0.5.

The outer loadings should be checked to analyze the individual reliability of the constructs, which should be greater than 0.707 according to Carmines and Zeller (72) or 0.55 according to Hwang et al. (73). Finaly, the multicollinearity analysis (variance inflation factor—VIF) helps to understand whether some observed variables explain the same construct as other observed variables. O'Brien (74) argues that a high and unacceptable value of VIF would be 10, and a low and therefore acceptable value would be less than 4, while Akinwande et al. (75) state that this value should not be higher than 5, and Marrucci et al. (65) argue that this value should not be higher than 3 (65). In this case, the SmartPLS 4 software used to run the structural model assumes multicollinearity problems with a VIF greater than 5 by default.

### 4.2 Structural model

This section includes the results of the SEM for active fans and non-active fans.

#### 4.2.1 The active fańs model

**Figure 3:**
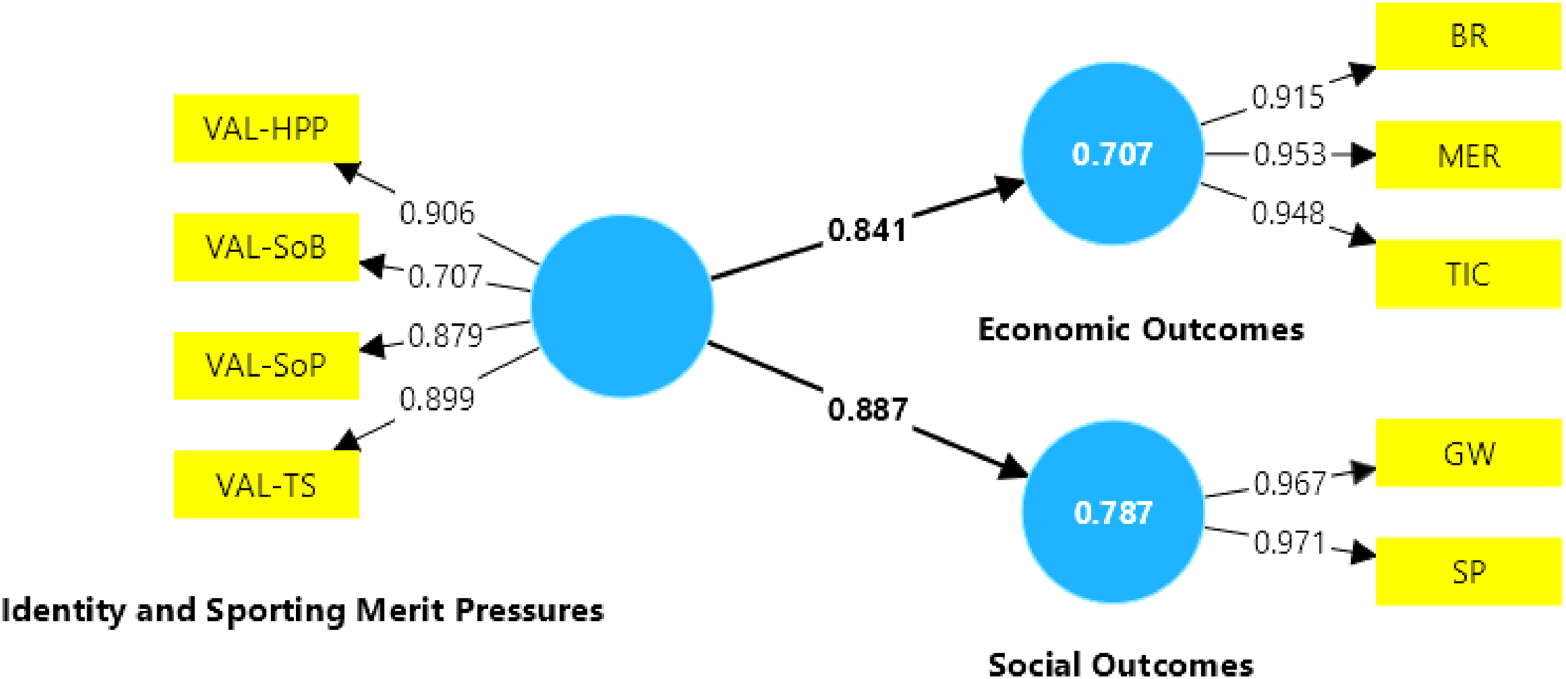
Results of the model with active fans.

The results of the SEM including active fans are shown in Figure 3. In this model, both the economic path coefficient (0.841) and the social path coefficient (0.887) are greater than 0.3. Therefore, identity and sporting performance pressures have a significant impact on economic and social outcomes. Similarly, the AVEs shown in Table 3 are greater than 0.5, so it can be concluded that the active fan model has internal consistency. As the Cronbanch is greater than 0.7 for each construct variable, it can be concluded that these construct variables are reliable.

**Table 3:** Results of the active fańs model constructs.

Constructs	Cronbach's alpha	Average variance extracted (AVE)
Economic Outcomes	0.933	0.881
Social Outcomes	0.935	0.939

Figure 3 shows that the R-squared for the economic outcomes (0.707) and social outcomes (0.787) are greater than 0.1, so the model is considered to have an adequate structural fit. Accordingly, all the indicators belonging to the construct variable of identity and sport performance pressure are reliable, as their outer loadings are equal or greater than 0.707. However, the observed variables with the highest values were VAL-HPP (high performing players) and VAL-TS (coaching staff). This suggests that active fans value the fact that the football club employs high performance players and high-performance coaching staff more than the stability of the playing squad (VAL-SoP) and the sense of belonging of the managers (VAL-SoB).

In terms of economic outcomes, the most highly rated variable was MER (merchandising), and in terms of social outcomes, the most highly rated variable was SP (sporting performance). An evaluation of multicollinearity through VIF is useful to understand if there are some variables that explain the same effects as another variable that belongs to the same construct variable. If this is the case, depending on the context of the exercise, the possibility of eliminating some of the variables that present this condition can be considered in order to obtain a better explanatory model.

Table 4 shows the multicollinearity analysis, which shows that the model with active fans does not present collinearity problems; however, the variables MER (merchandising) and TIC (ticket income) would be very close to collinearity. In this sense, it could be that the indicator that best explains the economic results is BR (broadcasting rights). Part of this explanation is related to the fact that active fans are composed of direct and indirect fans, and that direct fans value stadium attendance more, while indirect fans value following the club through broadcasting channels.

**Table 4:** Multicollinearity analysis for active fans.

Collinearity statistics	VIF (Original model)
BR	3.155
GW	4.369
MER	4.900
SP	4.369
TIC	4.467
VAL-HPP	3.097
VAL-SoB	1.456
VAL-SoP	2.613
VAL-TS	3.019

Regarding social outcomes, although GW and SP do not show collinearity problems, they have the same VIF value, which could indicate that they explain similar things, but GW was excluded because -active fans tend to be more focused on sporting performance, as these types of fans have a high affective attachment to football clubs. Therefore, it is necessary to verify this result in the context of active fans. Table 5 shows a comparison between these two model fits. The first model includes all variables (original model), and the second represents the model that includes only the BR (broadcasting rights) and SP (sporting performance) variables for economic and social outcomes respectively. According to Miles (70), the standardized mean square residual (SRMR) should be less than 0.1 and the normed fit index (NFI) should be greater than 0.9. In this sense, the model that includes only the BR and SP outcome variables provides a better fit.

**Table 5:** Model fitcomparison for active fans.

Model fit comparison	Model fit	Saturated model	Estimated model
Model with all indicators	SRMR	0.055	0.055
	NFI	0.864	0.864
Model only with BR and SP indicators	SRMR	0.057	0.060
	NFI	0.930	0.910

#### 4.2.2 The non-active fan's model

**Figure 4:**
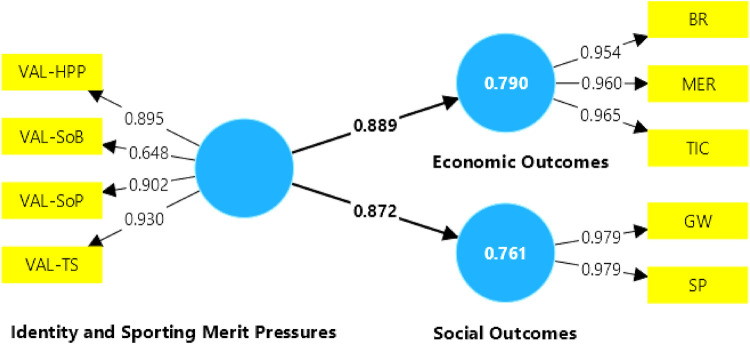
Model results with non-active fans.

The model for non-active fans supports the hypotheses that identity and sporting performance pressures influence both economic and social outcomes and is significant, as the path coefficients presented in Figure 4, 0.889 and 0.872 respectively, are greater than 0.3. Similarly, the Cronbach Alpha for both construct variables are greater than 0.7 and the AVEs are greater than 0.5, so the constructs are reliable and the model for nonactive fans has internal consistency (Table 6). Furthermore, for the economic and social outcomes, the R-squared, 0.790 and 0.761 respectively, are greater than 0.1, so the model is considered to have an adequate structural fit.

**Table 6:** Results of the non-active fańs model constructs.

Constructs	Cronbach's alpha	Average variance extracted (AVE)
Economic Outcomes	0.957	0.921
Social Outcomes	0.957	0.959

According to Figure 4, the variables with the highest outer loadings in the identity and sporting performance pressure construct were VAL-TS (coaching staff) and VAL-SoP (player stability), which means that non-active fans prefer these types of VAL-HPP (high performance players) and VAL-SoB (sense of belonging). Even the VAL-SoB variable is not reliable, as its outer loading value is lower than 0.707 (73). Therefore, the possibility of removing this variable from the model could be considered. For economic outcomes, TIC (ticket income) was the better rated variable in terms of outer loadings, and for social outcomes, GW (goodwill) and SP (sporting performance) have the same outer loading rating.

According to Table 7, in terms of economic results, the TIC, MER and BR variables show a high collinearity between them since their VIF value is higher than 5. Therefore, the two variables with the highest VIF (TIC and MER) are dropped from the mode. In terms of social outcomes, both GW and SP have the same VIF value, indicating that they explain similar aspects. This means that, statistically, either of them could be included or excluded, but SP was excluded because non-active fans tend to focus more on goodwill and image than on sporting performance, as these types of fans do not have a high affective attachment to football clubs. To address the multicollinearity issues, a new model was proposed that only included the BR and SP variables for sustainability outcomes and excluded the VAL-SoB variable, and the multicollinearity analysis was re-run, yielding a corrected VIF as shown in Table 7.

**Table 7:** Multicollinearity analysis for non-active fans.

Collinearity statistics	VIF (Original Model)	VIF (Corrected Model)
BR	5.214	1.000
GW	6.346	1.000
MER	5.873	—
SP	6.346	—
TIC	6.436	—
VAL-HPP	2.974	2.806
VAL-SoB	1.337	—
VAL-SoP	3.651	3.600
VAL-TS	4.870	4.870

Table 8 shows the comparison between two model fits. The first model refers to the full model, including the initial variables, and the second refers to the model with the corrected VIF, including only the BR and GW variables for the sustainability outcomes. Since the SRMR should be less than 0.1 and the NFI should be greater than 0.9, the corrected model that includes only the BR and GW outcome variables provides a better fit.

**Table 8:** Model fit comparison.

Model fit comparison	Model fit	Saturated model	Estimated model
Model with all indicators	SRMR	0.048	0.054
	NFI	0.881	0.870
Model only with BR and SP indicators	SRMR	0.037	0.039
	NFI	0.948	0.937

Summarizing the results of both models, considering active fans and non-active fans, Table 9 shows the main results in terms of statistical validation of hypothesis testing, construct variables and their respective observable variables.

**Table 9:** Summary of the results of the models.

Aspect for evaluation	Model with active fans	Model with non-active fans
Hypothesis testing	All hypotheses were significant. The highly rated was H2, which related identity and sporting performance pressures to social outcomes.	All were significant. The most highly rated was H1, which related identity and sport performance pressure to economic outcomes.
Identity and Sporting Performance Pressures	All variables were reliable. The most highly rated by active fans were VAL-HPP (high performance players) and VAL-TS (coaching staff).	All variables were reliable. The variables most valued by active supporters were VAL-TS (coaching staff) and VAL-SoP (player stability).
Economic outcomes	The outer loading indicated that the most relevant variable was MER (merchandising). However, due to a multicollinearity problem, the most relevant variable was BR (broadcasting rights)	The outer loading indicated that the most relevant variable was TIC (income from Single and Season Tickets). However, due to a multicollinearity issue, the most relevant variable was BR (broadcasting rights)
Social outcomes	The most relevant variable was SP (sport performance)	The most relevant indicator was GW (goodwill)

## 5 Discussion

This study is in line with previous research highlighting the need to balance commercial and sporting objectives in football club management (41, 58), as excessive economic exploitation can erode social capital, making it essential for clubs to engage stakeholders, especially fans, in value co-creation (17, 22). Football fans, driven by emotional connection and loyalty (9, 10), are no longer passive spectators but active participants who influence the club's revenue streams and reputation (27). This study contributes to the literature by linking fan-generated pressure to economic and social outcomes, a perspective that has previously been applied primarily to institutional and governmental stakeholders in European football (5–7). In contrast to previous studies, this research specifically examines how different fan typologies (active and non-active fans) exert pressure on the economic and social performance of clubs. Active fans, i.e., those who attend matches or follow the club via broadcasts, show a higher sense of urgency, especially with regard to sporting success and managerial decisions (29, 33, 59). In contrast, non-active fans, including passive and potential fans, are less emotionally engaged but can still contribute to club sustainability through indirect means such as broadcast revenues and goodwill (15, 16, 20, 27).

This study confirms that economic and social objectives are not mutually exclusive and that a well-managed club can achieve both by aligning its business strategy with the expectations of its fans. In terms of economic outcomes, merchandising and ticket sales were the most important revenue drivers for active fans, but broadcasting rights emerged as the most important factor for non-active fans. Given that European clubs derive substantial income from broadcasting, Colombian clubs must strengthen this revenue stream by developing more structured partnerships with streaming platforms and sports networks (17). In terms of social outcomes, the strongest driver for active fans was sporting performance, reinforcing the need for strategic recruitment of players and coaches to maintain engagement and revenues (20, 46). For non-active fans, the most valued social factor was goodwill and corporate image, highlighting the importance of community-driven initiatives, ethical management, and long-term reputation building. Football clubs need to recognize that fan expectations shape not only financial outcomes but also social credibility, reinforcing the need for balanced decision-making.

The findings of this study have theoretical relevance beyond Colombia, as identity-based and performance-based pressures are universal across football cultures (42). Applying this conceptual model to European football may reveal differences in fan behavior based on governance structures, club ownership models, and league commercialization. In addition, while this study is based on perceptions rather than quantitative market data, it provides valuable insights into fan motivation and engagement, which are critical for developing targeted marketing, pricing, and sustainability strategies. From a managerial perspective, football club executives must prioritize a long-term strategy that balances fan engagement, sporting success, and financial sustainability (17, 41). Investing in high-quality players and a stable coaching staff can improve performance and increase revenues from ticket sales, merchandise, and media rights. In addition, clubs should use fan data and direct engagement strategies, such as Club Atlético Nacional's fan census initiative, which conducted more than 50,000 effective surveys, to tailor loyalty programs and community engagement projects, such as inviting season ticket holders to experience the team by visiting the club's headquarters, taking photos with players, and giving a tour of the club's facilities.

The growing emphasis on social responsibility and sustainability requires clubs to engage in initiatives beyond the pitch, such as the promotion of women's football (36, 76), anti-racism campaigns (77), and environmental commitments (16, 35, 40). While Colombian clubs have begun to implement social and community programs, such as support for vulnerable groups and pet adoption, environmental sustainability initiatives, such as clean energy generation, are still in their infancy and represent an opportunity for future industry development. Therefore, football clubs need to recognize fans as co-creators of value and integrate their expectations into club strategies to ensure sustainable growth. By adopting data-driven engagement models, strengthening broadcast partnerships, and investing in both sporting and social programs, clubs can improve their competitive position and long-term financial stability.

## 6 Conclusions

This study underscores the importance of shifting football club management from a profit-driven approach to a stakeholder-inclusive model that values fan engagement. Fans play a crucial role in shaping both the economic and social outcomes of clubs through their emotional connection, loyalty and collective pressures. Our findings highlight that sporting identity and performance-related pressures, such as managerial commitment, player recruitment, coaching staff quality, and squad stability, have a significant impact on club sustainability.

Using a structural equation model (SEM), we analyzed how active and non-active fans influence the economic and social outcomes of clubs. Active fans, who attend matches or watch broadcasts, primarily value high-performing players and coaches, with broadcasting rights and sporting performance as their most important economic and social outcomes. Non-active fans, who are less involved, value the stability of the coaching staff, with broadcasting rights and goodwill as their main concerns.

While the proposed model confirms the importance of fan-generated pressure, it has limitations, particularly in terms of the representativeness of the sample. The high concentration of respondents from Medellín means that the results may not fully reflect the perspectives of all Colombian football fans. Future research should expand the geographic scope to analyze potential regional differences in fan behavior and compare findings across different football cultures. In addition, broader stakeholder involvement, including club executives, sponsors, and governing bodies, could provide a more comprehensive view of how different actors shape football governance.

## Data Availability

The datasets generated for this study are available upon request.
